# Maxillary Transverse Deficit: A Retrospective Study of Two Biologically Oriented Devices through a Digital Workflow

**DOI:** 10.3390/bioengineering9010031

**Published:** 2022-01-12

**Authors:** Graziano Montaruli, Simona Virgilio, Michele Laurenziello, Michele Tepedino, Domenico Ciavarella

**Affiliations:** 1 Department of Clinical and Experimental Dentistry, School of Dentistry, University of Foggia, 71121 Foggia, Italy; simonamontaruli04@gmail.com (S.V.); michele.laurenziello@unifg.it (M.L.); dipartimento.medicinaclinica@unifg.it (D.C.); 2 Department of Biotechnological and Applied Clinical Sciences, University of L’Aquila, 67100 L’Aquila, Italy; terapeuticaruvo@gmail.com

**Keywords:** maxillary transverse deficit, slow palatal expansion, rapid palatal expansion, digital workflow

## Abstract

The aim of this retrospective study was to compare the efficiency of two biologically oriented devices in achieving maxillary expansion: Rapid Palatal Expander (RPE) and Nitanium Palatal Expander-2 (NPE-2). Thirty-six subjects, divided in two equal groups, were included in this study. Maxillary dental arches were scanned using Trios 3 shape^®^, in order to perform a digital analysis of 3D models. The models were analyzed using Autodesk Fusion 360^®^ and Meshmixer^®^. All data obtained from analysis of pre-treatment and post-treatment models were processed using Prism^®^ software. The anterior arch width, the posterior arch width, the palate height, and palatal surface were measured to evaluate differences between the devices. A D’Agostino–Pearson normality test was done to check the data. A non-parametric *t*-test was used to compare the anterior and posterior arch width between the two groups, while a parametric *t*-test was used to compare the palatal height measurements between the two groups. The *p*-value was calculated. The limit value fixed was 0.05. Palatal width and surface showed a significant increase in both groups, but no significant changes in palatal height were found. The data processed showed that there were no significant differences between the devices (Δ*REP*−Δ*NPE*) in variation of anterior arch width, there were no significant differences in variation of posterior arch width and there were no significant differences in variation of palatal height. The comparison between the two groups showed that both methods were equally effective in correcting transverse defect.

## 1. Introduction

The rhino-maxillary complex is a connecting structure between the neurocranium and the splanchnocranium. It presents an intramembranous origin and its dislocation in space occurs through two processes: suture stress and periosteal remodeling guided by epigenetic factor [1].

Several factors are able to influence the etiopathogenesis of transversal anomalies [2]. Among them includes congenital alterations, dystrophies, metabolic disorders, infections, trauma as well bad habits, atypical swallowing, and oral breathing.

Transverse defects in growing patients can lead to significant changes in breathing efficiency.

Developing malocclusion occurs in dentoalveolar, skeletal, or mixed forms [3]. The transversal deficit is common to most malocclusions. Solving a skeletal or dentoalveolar transverse deficit is one of the first therapeutic goals to achieve [4].

The transverse deficits of the upper jaw often show an evident anomalous relation between the arches on the transverse plane in centric occlusion, with the development of a single or bilateral cross-bite [5]. The unilateral cross-bite causes an asymmetry of transverse and sagittal ratios as well as an alteration of normal condyle-fossa ratios [6,7].

Transverse deficiency can be corrected by a combined skeletal and dental expansion.

The skeletal expansion separates the right and the left maxillary halves at the median palatine suture; dental expansion results from movement of maxillary teeth.

Expansion devices can be classified as slow or rapid.

Nitanium^®^ Palatal Expander™ (NPE-2) (Ortho Organizers, Inc.—San Marcos, CA, USA) is a slow expansion appliance: A nickel-titanium temperature-activated single loop produces a light and continuous pressure on the palatine suture [8].

The increase in maxillary arch width in young children is due to a combination of opening of the palatine suture, tipping of the alveolar process, and molar tipping [9].

Skeletal expansion occurs simultaneously with dentoalveolar expansion in the transversal plane [10].

This appliance is capable of correcting molar rotation and requires no patient compliance [8,11]. An estimated 25% to 30% of all orthodontic patients can benefit from maxillary expansion and 85% of the class II cases can be improved by molar rotation, distalization, and expansion. The correction of molar rotation alone results in a net gain of space in the upper arch of more than 2 mm [12].

NPE-2 presents “shape memory” and “transition temperature”. The shape memory is the appliance’s property to return to its original shape after any deformation; the transition temperature is the appliance’s property to change its physical characteristics with changes in temperature [8,11].

Expansion is normally completed within two or four months, followed by a variable period of retention.

The Rapid Palatal Expander (RPE) is a rapid expansion appliance. It produces heavy forces at the sutural site that generate a massive hyalinization of the periodontium, which prevents dental movement. RPE appliance consists of an expansion screw that requires parents’ cooperation for the activation procedure.

The amount of skeletal or dental movement depends on the age of the patient during treatment. The median palatine suture experiences great morphological changes during growth [13]: The sutural growth can continue until it remains open and active, then it goes through a closing process [14,15].

The presence of bone bridges for more than 5% of the suture length determine unsurpassed mechanical resistance. The rapid maxillary expansion is therefore easier during childhood when the suture remains fibrous and poorly interdigitated [2].

The expansion carried out in the pre-puberal phase produces effects at both skeletal and dentoalveolar level which remain at the end of growth. The post-puberal expansion produces effects exclusively at the dentoalveolar level [2].

The rapid maxillary expansion offers a real gain of bone substance in cases of serious transverse deficits of the upper maxilla. The expansion of the upper maxilla also affects the intermaxillary, zygomatic-maxillary, inter-nasal, and maxillary-nose sutures [16].

Expansion is normally completed within two weeks. It follows a period of retention ranging from 4 to 6 months.

The orthopedic expansion is able to solve a transversal defect and to increase and improve the patient’s breathing capacity.

The aim of this study was to describe and to compare the efficiency of RPE and NPE-2 appliances in achieving maxillary expansion by a digital workflow. The anterior and posterior arch width, the palatal height, and palatal surface were quantified to learn about advantages and differences between these appliances.

## 2. Materials and Methods

The sample consisted of 36 patients treated with RPE or NPE-2 appliances at the Orthodontics and Sleep Medicine Unit of the Dental Clinic of the University of Foggia. The specific indications by Declaration of Helsinki and the Guiding Principles in the Care and Use of Animals (DHEW Publication, NIH, 80-23) were respected with Protocol Number 43/CE/2019.

The patients included in this retrospective study, in mixed dentition, presented a mild to severe transverse maxillary deficiency and required palatal expansion as part of their comprehensive orthodontic treatment. These exclusion criteria were applied: patients with absence of permanent first molar, and morphologic alterations of the permanent first molars which hindered the positioning of a molar band.

Written informed consent was obtained from the patients and their parents.

The patients were divided into two equal groups of 18 patients. Group 1 was treated with an NPE-2 device, while Group 2 was treated with a RPE device. Group 1 included the patients in mixed dentition with molar rotation who were treated with the Nitanium Palatal Expander. Group 2 included the patients in mixed dentition who were treated with Rapid Palatal Expander.

In Group 1, the mean age before treatment (T0) was 11.7 years ± 2 and at the end of the expansion therapy (T1) it was 12.3 years ± 2.1. In Group 2, the mean age at T0 was 9.8 years ± 1.7 and 10.4 years ± 1.7 at T1.

The mean duration of therapy (T1−T0) was:

NPE-2: 5 months ± 2;

RPE: 5 months ± 1.

T0 and T1 records included maxillary and mandibular casts, panoramic radiograph, occlusal radiograph, lateral and postero-anterior radiographs, photographs.

A cephalometric analysis was performed at T0 and at T1.

Expansion was considered adequate when the palatal cusp of the maxillary posterior teeth contacted the buccal cusp of the mandibular posterior teeth. The amount of over-expansion was planned to compensate for relapse after expansion.

**Figure 1:**
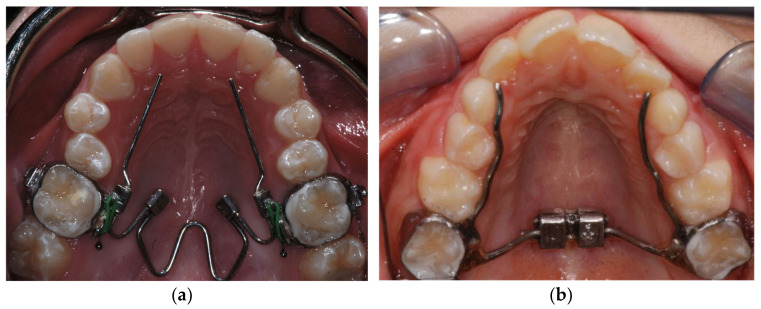
(**a**) Nitanium^®^ Palatal Expander™ (NPE-2); (**b**) Rapid Palatal Expander (RPE).

Nitanium^®^ Palatal Expander™ is a fixed-removable device with a temperature-activated coffin loop that is bilaterally connected by lingual sheaths to the maxillary molar bands; it presents two arms (Orthology 0.036 in diameter) extended from the bands to the premolars/canines in order to obtain an anterior expansion (Figure 1a). It also presents two stainless steel omega loops which are the only components that can be activated by the clinician.

The appliance is manufactured in different sizes; the proper one is selected using the formula (dUPS-3) + (dLCP-dUMPC). Where dUPS is the distance between upper palatal surfaces; dLCP is the distance between lower central fossae; dUMPC is the distance between upper mesial-palatal cusps measured on the casts; and 3 represents the thickness, expressed in millimeters, of the bands, and of the lingual tubes applied to the bands.

Before insertion, the NPE-2 has to be treated with a cold spray or locked by a steel ligature for an easy placement of the device. Once the temperature increases in the oral cavity the Nitanium^®^ recovers its stiffness and starts to exert the force that produces the palatal expansion [11].

The Rapid Palatal Expander is a fixed device that consists of an expansion screw activated using a key [17,18,19,20], two bands placed on the first maxillary molars and connected to the screw by two stainless steel arms. Two other arms are modeled to reach the premolars, in order to transfer the force also to the anterior region of the palate (Figure 1b). The patients’ parents were instructed to activate the screw twice a day until correction of the transverse occlusal relationship was achieved. This outcome was verified by the clinicians.

### 2.1. Models Evaluation

For each patient of both groups, maxillary dental casts were collected at T0 and T1, and scanned using Trios 3 shape^®^ (3 Shape Inc., Copenhagen, Denmark) to obtain digital models.

The 3D models were analyzed using Autodesk Fusion 360^®^ and Autodesk Meshmixer^®^ (San Rafael, CA, USA) computerized programs.

Autodesk Fusion 360^®^ software was used to record palatal height, anterior width, and posterior width. These variables were recorded using the “Inspect-Measure” function: For each variable the proper landmarks were identified and selected on the right and on the left. This function quantified the distance between the landmarks.

The anterior arch width represented the distance between the deepest points of the transverse grooves of the first premolars.

The posterior arch width was measured between the permanent first molars using two methods: The posterior width according to Pont, i.e., the distance between the right and left point at the intersection between the transverse groove and the buccal grooves; the posterior width according to McNamara, i.e., the distance between the points where lingual grooves meet gingival edges of the first molars.

**Figure 2:**
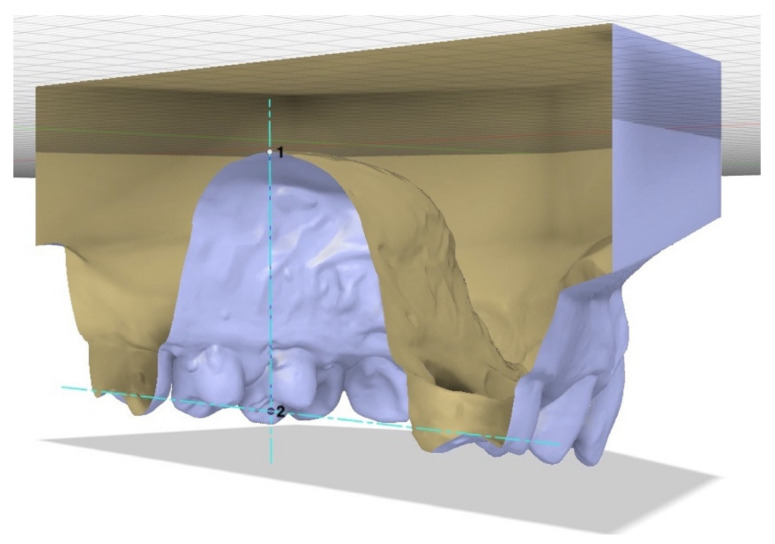
The palatal height is the perpendicular to the median plane of raphe drawn from palatal surface (point **1**) up to the level of occlusal plane (point **2**).

The palatal height was defined as the line perpendicular to the median plane of raphe, drawn from the palatal surface to the occlusal plane. To estimate this variable, it was necessary to define two planes: the median plane of raphe and a plane passing through the right and left point at the intersection between the transverse groove and the buccal grooves of the permanent first molars and perpendicular to the median plane of raphe. Then the height could be recorded with the same function as width, measuring the distance between palatal surface, and occlusal plane along the points where the two plans intersect (Figure 2).

**Figure 3:**
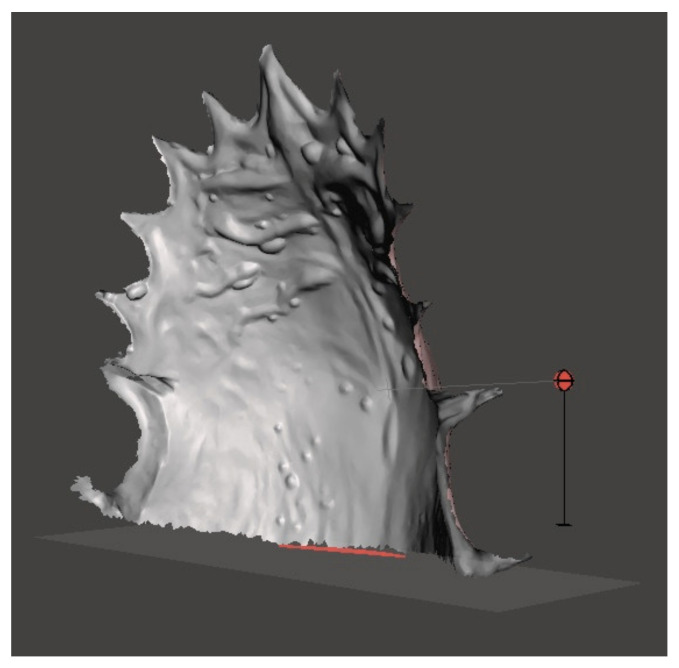
The palatal surface separated from entire model.

Meshmixer^®^ software was used to measure the palatal surface, defined as the surface bounded by the palatal–gingival contour of teeth from the first right molar to the first left molar and by a line connecting the distal surfaces of first molars. The palatal surface was selected with the “Select-Brush” function, then isolated from the entire model to be measured (Figure 3).

Data obtained from analysis of pre-treatment and post-treatment models of all the patients in both groups were appropriately collected and subjected to statistical analysis.

### 2.2. Statistical Analysis

All data, obtained from analysis of pre-treatment and post-treatment models, were processed using Prism^®^ software (San Diego, CA, USA).

First, a D’Agostino–Pearson normality test was done to check if data for each variable had a normal distribution or not, and to evaluate if parametric or non-parametric tests would be needed.

Then, the null hypothesis that there would be no significant changes associated with the use of the RPE, nor with NPE-2, was tested. In addition, to determine the presence of differences between the two groups, the null hypothesis that there would be no significant differences between treatments was tested.

Type error was set as *p* < 0.05 for all the tests.

## 3. Results

The first research question concerned the efficacy of both devices testing the null hypothesis that there would have been no significant changes associated with treatment (Table 1).

Patients treated with RPE presented before treatment an anterior arch width of 32.54 ± 7.28 mm, a posterior arch width of 45.57 ± 1.01 mm (Pont’s landmarks), and a palatal height of 13.53 ± 2.07 mm. At the end of treatment an anterior arch width of 36.79 ± 7.91 mm, a posterior arch width of 49.72 ± 2.32 mm, and a palatal height of 13.15 ± 3.4 mm were measured.

By analyzing each parameter individually, we observed an increase in anterior arch width of 4.25 mm, an increase in posterior arch width of 4.15 mm, and a reduction in palatal height of 0.38 mm.

The values of anterior and posterior arch width before and after treatment showed a normal distribution according to D’Agostino-Pearson normality test. Parametric *t*-tests showed a *p*-value < 0.0001, clearly smaller than the limit value of 0.05, so the null hypothesis was rejected for RPE treatment.

On the other hand, palatal height did not show a normal distribution. The non-parametric *t*-test showed a *p*-value = 0.9197 and the null hypothesis could not be rejected: there were no significant changes associated with the use of RPE regarding palatal height.

Patients treated with NPE-2 presented before treatment an anterior arch width of 34.54 ± 2.94 mm, a posterior arch width of 46.01 ± 2.1 mm (Pont’s landmarks), and a palatal height of 17.24 ± 5.25 mm. At the end of treatment an anterior arch width of 39.31 ± 2.26 mm, a posterior arch width of 50.45 ± 1.8 mm, and a palatal height 17.23 ± 2.37 mm were recorded.

By analyzing each parameter individually, we observed an increase in anterior arch width of 4.64 mm, an increase in posterior arch width of 4.44 mm, and a reduction in palatal height of 0.01 mm.

All the variables showed a normal distribution according to the D’Agostino–Pearson normality test. Parametric *t*-tests for anterior and posterior arch width showed a *p*-value <0.0001, so the null hypothesis was rejected: There were significant changes associated with the use of NPE-2. The parametric *t*-test for palatal height showed a *p*-value of 0.987 and the null hypothesis could not be rejected: There were no significant changes associated with the use of NPE-2.

After analyzing the efficacy of the devices in both groups, the results obtained were compared to understand any differences in treatment testing the null hypothesis that there are no significant differences between different types of treatment (Table 2).

A non-parametric *t*-test was used to compare the anterior and posterior arch width between the two groups, while a parametric *t*-test was used to compare the palatal height measurements between the two groups.

**Figure 4:**
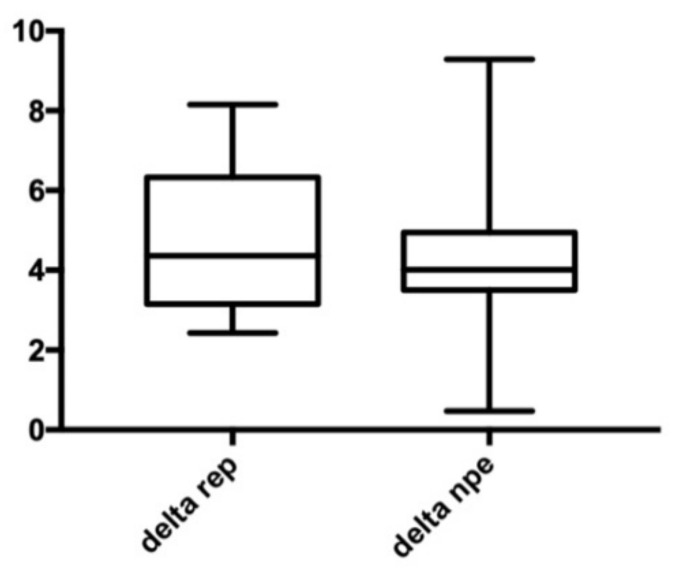
Anterior width. Delta REP is the variation in patients treated with RPE; Delta NPE-2 is the variation in patients treated with NPE-2; the numbers show the variation measured in millimeters.

**Figure 5:**
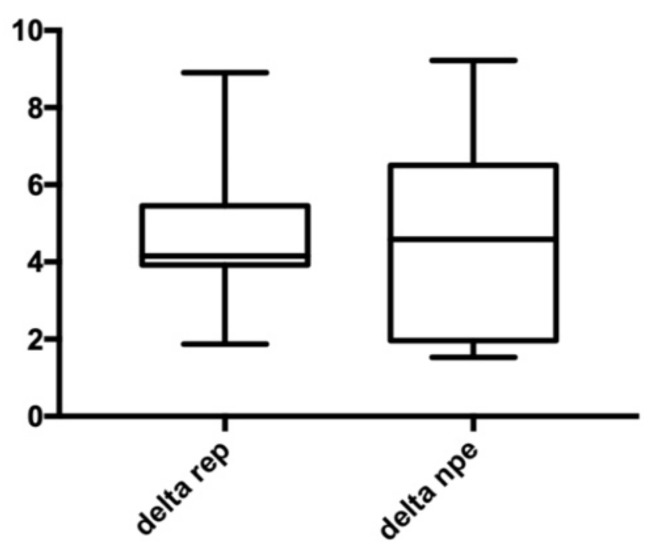
Posterior width. Delta REP is the variation in patients treated with RPE; Delta NPE-2 is the variation in patients treated with NPE-2; the numbers show the variation measured in millimeters.

**Figure 6:**
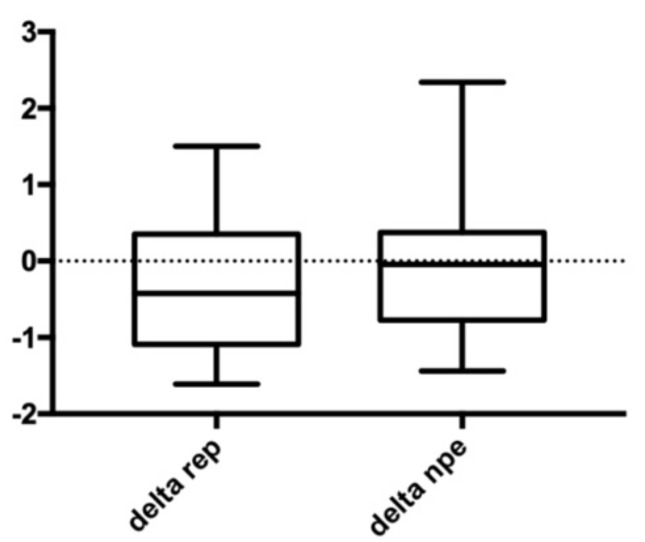
Palatal height. Delta REP is the variation in patients treated with RPE; Delta NPE-2 is the variation in patients treated with NPE-2; the numbers show the variation measured in millimeters.

Our results showed that there were no significant differences in the variation of anterior arch width (Figure 4), no significant differences in variation of posterior arch width (Figure 5), and no significant differences in variation of palatal height (Figure 6). Therefore, the null hypothesis was accepted.

After the analysis of posterior arch width measured with Pont’s landmarks, an analysis of posterior arch width measured with McNamara’s landmarks was done (Table 3).

Patients treated with RPE presented a posterior arch width (McNamara) of 34.69 ± 2.65 mm before treatment, while at the end of treatment it was 38.54 ± 2.82 mm. The increase after therapy was 3.84 mm.

On the other hand, patients treated with NPE-2 presented before therapy a posterior arch width (McNamara) of 35.02 ± 2.37 mm and at the end of treatment it was 38.95 ± 2.55 mm. The increase after therapy was 3.92 mm.

The values of posterior arch width (McNamara) in both groups had a normal distribution according to D’Agostino–Pearson normality test. The parametric *t*-test showed in the group treated with RPE a *p*-value of 0.0002 and in the group treated with NPE-2 a *p*-value <0.0001: there were significant changes of this value using both devices.

Nevertheless, it is important to understand the difference between posterior arch width according to Pont and posterior arch width according to McNamara. Subtracting from posterior arch width according to Pont the one according to McNamara, before and after treatment, it is possible to understand how this difference changes during therapy (Table 4).

The difference between these parameters after treatment showed an increase of 0.31 mm in patients treated with RPE and 0.52 mm in patients treated with NPE-2. In both cases this difference was not significant.

After analysis of linear values (width and height), a similar evaluation of palatal surface was done (Table 5).

Patients treated with RPE presented before treatment a palatal surface of 1455 ± 159.7 mm^2^, while at the end of treatment it was 1623 ± 157.3 mm^2^. The increase after therapy was 168 mm^2^.

Patients treated with NPE-2 presented before treatment a palatal surface of 1560 ± 156.9 and of 1693 ± 131.6 mm^2^ at the end of treatment. The increase after therapy was 133 mm^2^.

Palatal surface data in both groups of patients had a normal distribution according to D’Agostino–Pearson normality test. Parametric *t*-tests showed in the group treated with RPE a *p*-value of 0.0032 and in the group treated with NPE-2 a *p*-value of 0.0096. There were significant changes of this value using both devices.

The comparison of this parameter’s increases during therapy in both groups (Table 6) showed that there were no significant differences between patients treated with RPE and patients treated with NPE-2. The changes in palatal surface during treatment with both devices (ΔNPE-2 and ΔRPE) had a normal distribution according to D’Agostino–Pearson normality test. Parametric *t*-tests showed a *p*-value of 0.19.

## 4. Discussion

The knowledge on the rapid and slow palatal expansion is by now dated and supported by endless literature. It is always interesting, however, to understand the changes in other kinds of expansion in terms of bone and dentoalveolar results.

In patients affected from unilateral or bilateral posterior cross-bite, a comparison of dental and dentoalveolar changes between RPE and NiTi palatal expander was made. The substantial findings, from which we started our research, were: RPE always opened the suture while NiTi palatal expander in 85.4% of cases with an excessive tipping (11.69 vs. 6.08°). The molar rotation with NiTi palatal expander was 26.61 vs. 1.58° with RPE, with intermolar width of, respectively, 4.76 vs. 6.26 mm [21].

In order to analyze three-dimensional effects of stress distribution and displacement on the craniofacial structures, an analysis of three-dimensional effects of NPE-2 and Hyrax appliance in early mixed dentition using finite element analysis was made [22].

NPE-2 and Hyrax produced similar stress patterns in the early mixed dentition period. According to this finite element method study, NPE2 is equally effective as Hyrax when used in early mixed dentition period as it exhibits orthopedic nature of expansion with minimal residual stresses in the craniofacial structures.

Another comparative study was done using a finite element model of a young maxillary bone [23]. The stress distribution patterns within the maxillary complex during the expansion by a slow maxillary expansion screw and NPE- 2 was analyzed.

The results of this study suggested that NPE-2, even if it is an orthodontic device, is capable of producing mild to moderate orthopedic changes in maxilla.

In accordance with the above studies, in the present one changes in anterior and posterior arch widths occurred (according to Pont) are the evidence of skeletal and dental expansion produced by these appliances.

These parameters in both groups showed a significant increase: All this means that both RPE method (<0.0001) and NPE-2 method (<0.0001) are effective in the treatment of transverse defects.

During expansion as width increases, the palatal height decreases. In this retrospective study, no significant changes in the palatal depth were found. It is possible that an increase in the dentoalveolar height, caused by teeth’s eruption [24] and a lowering of palatal shelves [25], offset each other and resulted in no significant changes in palatal depth.

The comparison between the two groups showed that both methods are equally effective in correcting transverse defect.

A limit of the measurements is represented by landmarks’ position for data collection. They are detected on the occlusal face of premolars and molars, so the measurements are affected by dental rotations or tipping. On the other hand, with this method, we know all the expansion, but we do not know how much expansion is skeletal and how much dental.

This is the reason why it is necessary to analyze another parameter: the posterior arch width according to McNamara. Measuring this value, it is possible to know only the orthopedic expansion.

It is very important to know the difference between the posterior arch width according to Pont and the one according to McNamara. The greater this difference (Pont–McNamara), the more the device will have a dental effect (dental torque’s variation). The smaller this difference, the more the device will have a skeletal effect.

According to several studies, in patients treated with rapid expansion, after two weeks an increase in width due to skeletal growth by 80% and to teeth’s movement by 20% was detected.

During the following weeks bone filled the suture space and skeletal expansion decreased. However, the whole expansion did not change offset by tooth movement.

Initially with the RPE method there was a predominantly orthopedic action with minimal orthodontic movement, while in the following weeks there was an increase in dental expansion at the expense of skeletal expansion.

In patients treated with slow expander, however, the increase in width was from the beginning 50% orthodontic and 50% orthopedic.

In our study the linear analysis of posterior width showed that torque variation in posterior dental elements is greater in the group treated with NPE-2, although this difference is not significant. However, it is not possible to establish what causes this difference: The hypothesis is that it may be the type of force applied.

The analysis of palatal area shows orthopedic expansion, similarly to McNamara index, not affected by any variations in dental torque. This analysis confirms what has been demonstrated with other variables: an increase in palatal surface was 11.55% in the group treated with RPE and 8.53% in group treated with NPE-2.

Nevertheless, all these results have to be confirmed by larger clinical studies in order to evaluate more closely the performance of NPE-2 versus a rapid palatal expander in terms of skeletal and dentoalveolar defects’ correction.

Finally, we have to highlight the importance of virtual planning in order to obtain an ideal occlusion.

In relation to this topic, the computerized occlusal analysis system has to be considered the better occlusal indicator when compared with other non-digital indicators available [26].

In addition, a modified technique for evaluating static occlusal contacts may improve the accuracy in determining occlusal contacts and facilitate occlusal adjustments procedures [27].

Although at present many clinicians treat this malocclusion using TADs (Temporary Anchorage Devices) which provide a skeletal anchor reducing dental effects [28], it is still recommended to use a Rapid Palatal Expander with dental anchors in deciduous or early mixed dentition.

Patients in permanent dentition can be successfully treated with a slow expansion (NPE-2 or other similar devices) trying to reduce stress on molars’ roots or with skeletal anchoring devices.

## 5. Conclusions

Based on the findings of this retrospective study, the following conclusions were drawn:

1. The expansion of rhino-maxillary complex is an effective method for treating transverse maxillary defects;
2. The comparison of groups of patients treated with Rapid Palatal Expander and Nickel–Titanium Palatal Expander showed that both methods are equally effective in treating the transverse defect;
3. The choice of one device in relation to the other should be made by carefully assessing the age of the patient and the type of dentition present;
4. The results of NPE-2 in terms of skeletal or dental expansion areS not predictable.

## Figures and Tables

**Table 1:** Mean values of anterior width, posterior width (Pont’s landmarks), and palatal height before and after treatment in patients treated with RPE or NPE-2 (millimeters).

Mean (mm)	Pre-T				Post-T				Δ(Post-T–Pre-T)		Normality Test		Unpaired Non-Parametric *t*-Test Kolgomorov–Smirnov		Unpaired Parametric *t*-Test	
	RPE		NPE-2		RPE		NPE-2		RPE	NPE-2	RPE	NPE-2	RPE	NPE-2	RPE	NPE-2
	Mean	SD	Mean	SD	Mean	SD	Mean	SD								
Anterior width	32.54	7.28	34.54	2.94	36.79	7.91	39.31	2.26	4.25	4.64	yes	yes			<0.0001 *	<0.0001 *
Posterior width (Pont)	45.57	1.01	46.01	2.1	49.72	2.32	50.45	1.8	4.15	4.44	yes	yes			<0.0001 *	<0.0001 *
Palatal height	13.53	2.07	17.24	5.25	13.15	3.4	17.23	2.37	−0.38	−0.01	no	yes	0.9197 ^ns^			0.987 ^ns^

SD = standard deviation; T = treatment; ns = not statistically significant; * *p* < 0.05 = statistically significant.

**Table 2:** Changes in frontal width, posterior width (Pont’s landmarks), and palatal height before treatment and after treatment in patients treated with NPE-2 and RPE.

Mean (mm)	Δ(Post-T–Pre-T)		Normality Test	Unpaired Non-Parametric *t*-Test Kolgomorov–Smirnov	Unpaired Parametric *t*-Test
	RPE	NPE-2			
Anterior width	4.25	4.64	no	0.6279 ^ns^	
Posterior width	4.15	4.44	no	0.8572 ^ns^	
Palatal height	−0.38	−0.01	yes		0.4801 ^ns^

T = treatment; ns = not statistically significant.

**Table 3:** Mean values of posterior width (McNamara’s landmarks) in patients treated with RPE and NPE-2.

Mean (mm)	Pre-T		Post-T		Δ(Post-T–Pre-T)	Normality Test	Unpaired Non-Parametric *t-*Test Kolgomorov–Smirnov	Unpaired Parametric *t*-test
	Mean	SD	Mean	SD				
RPE	34.69	2.65	38.54	2.82	3.84	yes		0.0002 *
NPE-2	35.02	2.37	38.95	2.55	3.92	yes		<0.0001 *

SD = standard deviation; T = treatment; * *p* < 0.05 = statistically significant.

**Table 4:** Mean value of difference between posterior width according to Pont and posterior width according to McNamara.

Pont–McNamara (mm)	Pre-T		Post-T		Δ(Post-T–Pre-T)	Normality Test	Unpaired Non-Parametric *t-*Test Kolgomorov–Smirnov	Unpaired Parametric *t*-Test
	Mean	SD	Mean	SD				
RPE	10.88	1.06	11.19	1.41	0.31	no	0.49 ^ns^	
NPE-2	10.99	0.92	11.51	0.96	0.52	yes		0.11 ^ns^

SD = standard deviation; T = treatment; ns = not statistically significant.

**Table 5:** Mean values of palatal surface before and after treatment in patients treated with RPE and NPE-2.

Surface (mm^2^)	Pre-T		Post-T		Δ(Post-T–Pre-T)	Normality Test	Unpaired Non-Parametric *t*-Test Kolgomorov–Smirnov	Unpaired Parametric *t*-Test
	Mean	SD	Mean	SD				
RPE	1455	159.7	1623	157.3	168	yes		0.0032 *
NPE-2	1560	156.9	1693	131.6	133	yes		0.0096 *

SD = standard deviation; T = treatment; ns = not statistically significant; * *p* < 0.05 = statistically significant.

**Table 6:** Changes in the palatal area in patients treated with RPE and NPE-2 during treatment.

Surface (mm^2^)	RPE	NPE-2	Normality Test	Unpaired Non-Parametric *t*-Test Kolgomorov–Smirnov	Unpaired Parametric *t*-Test
Δ(Post-T–Pre-T)	167.4	132.5	yes		0.19 ^ns^

T = treatment; ns = not statistically significant.

## Data Availability

The data presented in this study are available on request from corresponding author. The data are not publicly available due to patients’ privacy.
